# Learning to propagate labels on graphs: An iterative multitask regression framework for semi-supervised hyperspectral dimensionality reduction

**DOI:** 10.1016/j.isprsjprs.2019.09.008

**Published:** 2019-12

**Authors:** Danfeng Hong, Naoto Yokoya, Jocelyn Chanussot, Jian Xu, Xiao Xiang Zhu

**Affiliations:** a Remote Sensing Technology Institute (IMF), German Aerospace Center (DLR), Wessling, Germany; b Signal Processing in Earth Observation (SiPEO), Technical University of Munich (TUM), Munich, Germany; c Geoinformatics Unit, RIKEN Center for Advanced Intelligence Project (AIP), RIKEN, Tokyo, Japan; d Univ. Grenoble Alpes, CNRS, Grenoble INP, LJK, Grenoble, France

**Keywords:** Dimensionality reduction, Graph learning, Hyperspectral image, Iterative, Label propagation, Multitask regression, Remote sensing, Semi-supervised

## Abstract

Hyperspectral dimensionality reduction (HDR), an important preprocessing step prior to high-level data analysis, has been garnering growing attention in the remote sensing community. Although a variety of methods, both unsupervised and supervised models, have been proposed for this task, yet the discriminative ability in feature representation still remains limited due to the lack of a powerful tool that effectively exploits the labeled and unlabeled data in the HDR process. A semi-supervised HDR approach, called iterative multitask regression (IMR), is proposed in this paper to address this need. IMR aims at learning a low-dimensional subspace by jointly considering the labeled and unlabeled data, and also bridging the learned subspace with two regression tasks: labels and pseudo-labels initialized by a given classifier. More significantly, IMR dynamically propagates the labels on a learnable graph and progressively refines pseudo-labels, yielding a well-conditioned feedback system. Experiments conducted on three widely-used hyperspectral image datasets demonstrate that the dimension-reduced features learned by the proposed IMR framework with respect to classification or recognition accuracy are superior to those of related state-of-the-art HDR approaches.

## 1 Introduction

Recently, hyperspectral imaging in sensing techniques has garnered growing attention for many remote sensing tasks (Plaza et al., 2009), such as land-use and land-cover classification (Yu et al., 2017, Gan et al., 2018, Hang et al., 2019), large-scale urban or agriculture mapping (Dell’Acqua et al., 2004, Yang et al., 2013, Fan et al., 2015, Xie and Weng, 2017), spectral unmixing (Henrot et al., 2016, Hong et al., 2017, Zhong et al., 2016, Hong et al., 2019a), object detection (McCann et al., 2017, Wu et al., 2018, Li et al., 2018, Wu et al., 2019), and multimodal scene interpretation (Tuia et al., 2016, Yokoya et al., 2018, Zhu et al., 2019, Liu et al., 2019), as forthcoming spaceborne spectroscopy imaging satellites (e.g., EnMAP (Guanter et al., 2015)) make hyperspectral imagery (HSI) available on a larger scale. Although HSI features richer spectral information than RGB (Kang et al., 2018) and multispectral (MS) data (Hong et al., 2015), yielding more accurate and discriminative detection and identification of unknown materials, yet the very high dimensionality in HSI also introduces some crucial drawbacks that need to be taken seriously: high storage cost, information redundancy, and the performance degradation resulting from *the curse of dimensionality*, to name a few. A general but effective solution to these issues is *dimensionality reduction*, also referred to as *subspace learning*. In this process, we expect to compress the HSI to a low-dimensional subspace along the spectral dimension while preserving the highest possible spectral discrimination.

With the significant support in both theory and practice as well as a fact that the learning-based strategy is somehow superior to the manually-designed feature extraction (Hong et al., 2016a), a considerable number of subspace learning approaches have been designed and applied to hyperspectral data processing and analysis in the past decades (Licciardi et al., 2009, Huang and Yang, 2015, Hong et al., 2016b, Luo et al., 2016, Liu et al., 2017, Xu et al., 2018a, Xu et al., 2019), particularly hyperspectral dimensionality reduction (HDR) (Gao et al., 2017a, Hong et al., 2017, Gao et al., 2017b) and spectral band selection (Sun et al., 2015, Sun et al., 2017a). Depending on their different learning strategies, HDR techniques are roughly categorized as unsupervised, supervised, or semi-supervised strategies.

The classic principal component analysis (PCA) (Martínez and Kak, 2001) is a user-friendly dimensionality reduction method for that is limited to capturing the underlying topology of the data. Rather, manifold learning techniques (e.g., locally linear embedding (LLE) (Roweis and Saul, 2000), Laplacian eigenmaps (LE) (Belkin and Niyogi, 2003), local tangent space alignment (LTSA) (Zhang and Zha, 2004), and their variants: locality preserving projections (LPP) (He and Niyogi, 2004), neighborhood preserving embedding (NPE) (He et al., 2005), large-scale LLE (Hong et al., 2016c), enhanced-local tangent space alignment (ENH-LTSA) (Sun et al., 2014)), by and large, follow the graph embedding framework presented in Yan et al. (2007). This framework starts with the construction of graph (topology) structure and aim at learning a low-dimensional data embedding while preserving the topological structure. Some popular and advanced methods have been proposed based on the graph embedding framework for HDR. For example, Ma et al. (2010) proposed to locally embed the intrinsic structure of the hyperspectral data into a low-dimensional subspace for hyperspectral image classification. Li et al. (2012) modeled the locally neighboring relations between hyperspectral data in a linearized system for HDR. In Huang et al. (2019), a multi-feature manifold discriminant analysis was developed on the basis of graph embedding framework for hyperspectral image classification. Authors of Sun et al. (2014) upgraded the existing landmark isometric mapping approach for the fast and nonlinear HDR. The same investigators (Sun et al., 2017b) further extended their work to linearly extract the low-dimensional representation with sparse and low-rank attribute embeddings for HSI classification. In Hong et al. (2017), a joint spatial-spectral manifold embedding is developed to extract the discriminative dimension-reduced features. Subsequently, Huang et al. (2019) proposed a general spatial-spectral manifold learning framework to reduce the dimension of hyperspectral imagery.

In supervised HDR strategies, the main consideration is the discrimination between intra-class and inter-class, where different discriminative rules are followed: local discriminative analysis (LDA) (Martínez and Kak, 2001), local fisher discriminative analysis (LFDA) (Sugiyama, 2007), sparse discriminant analysis (Huang and Yang, 2015), noise-adjusted discriminant analysis (Li et al., 2013), feature space discriminant analysis (Imani and Ghassemian, 2015), and so on. Despite the superior class separability, these methods still might fail to robustly represent the features due to sensitivity to various complex noises and ill-conditioned statistical assumptions, especially in the case of small-scale samples. Unlike the aforementioned approaches that seek to project the original data directly into a discriminative subspace, Ji and Ye (2009) simultaneously performed dimensionality reduction and classification under a regression-based framework, in order to find an optimally latent subspace where the decision boundary is expected to be better determined. With the local manifold regularization in the projected subspace, this strategy has been successfully applied and extended to learn the discriminative representation for supervised HDR (Hong et al., 2018).

Most previously-proposed HDR methods adhere to either the unsupervised or the supervised strategy, yet the labeled and unlabeled information is less frequently taken into consideration. A straightforward way to consider the unlabeled samples is the graph-based label propagation (GLP) (Zhu et al., 2003), which has been successfully applied to semi-supervised HSI classification (Li et al., 2016) together with the support vector machine (SVM) classifier. To effectively improve the discrimination and generalization of dimension-reduced features, some proposed semi-supervised HDR works have been proposed by the attempt to preserve the potentially global data structure that lies in the whole high-dimensional space. For example, Ma et al. (2015) followed a graph-based semi-supervised learning paradigm for HDR and classification, where the graphs are constructed by different local manifold learning approaches. A general but effective work integrating LDA with LPP, called semi-supervised local discriminant analysis (SELD), was proposed in Liao et al. (2013) for a semi-supervised hyperspectral feature extraction.Inspired by GLP, (Zhao et al., 2014) enhanced the performance of LDA by jointly utilizing the labels and “soft-labels” predicted by GLP for the semi-supervised subspace dimensionality reduction. Wu and Prasad (2018) proposed a similar approach to achieving a semi-supervised discriminative dimensionality reduction of HSI by embedding pseudo-labels (instead of the similarity measurement in LPP (Liao et al., 2013)) into LFDA rather than LDA in Zhao et al. (2014).

### 1.1 Motivation and objectives

Although these proposed semi-supervised approaches have been proven to be effective in handling the issue of HDR to some extent, yet their graph structures for unlabeled samples are constructed either from the similarity measurement (e.g., using RBF) or from the pseudo-labels inferred by GLP or pre-trained classifier. The resulting features by using this type of graph construction strategy is neither robust nor generalized, due to the noisy data and labels as well as the scarce labeled samples. Also, these semi-supervised algorithms, as often as not, attempt to find a single transformation that connects the original data and the subspace to be estimated. On account of the complexity in the learning process, the optimal subspace search is hardly accomplished only by a single transformation. On the other hand, in spite of being guided by label information, there is still lack of an explicit and direct connection between the learned subspace and the label space in the subspace learning strategy interpreted by a single projection, further causing the performance bottleneck. In addition, these subspace-learning-based models are commonly treated as a disjunct feature learning step before classification. In other words, it is unknown what kinds of features in the learning process may be capable of improving classification accuracy.

According to these factors, our objectives in this paper can be summarized as follows: 1) to bridge the to-be-estimated subspace with the label information more explicitly and effectively; 2) to introduce many unlabeled samples for improving the model’s generalization ability; 3) and to refine the quality of class indicators of unlabeled samples for high discriminative HDR.

### 1.2 Method overview and contributions

Towards the aforementioned goals, a novel regression-induced learning model motivated by the joint learning (JL) framework (Ji and Ye, 2009, Hong et al., 2018) is proposed, which seeks to learn an optimal subspace by considering the correspondences between the training samples and labels on a to-be-estimated latent subspace. We further extend the JL framework to a multitask regression model with the joint embedding of labeled and unlabeled samples. In the multitask framework, we also propose to adaptively learn a *soft-graph* structure from the data rather than utilizing a *hard-graph* (fixed graph) constructed manually or generated by additional algorithms, yielding a high-performance and more generalized label propagation. In the meantime, to facilitate the use of pseudo-labels more effectively, the learned graph can be updated after each outer iteration ends, and the pseudo-labels accordingly refined, thereby enabling the learned features to be progressively optimized. More specifically, the main contributions of this work can be highlighted as follows.

• We propose a JL-based variant: a novel iterative multitask regression (IMR) framework by simultaneously considering few labeled samples and unlabeled samples in quantity, with the application to semi-supervised HDR.
• We adaptively learn the connectivity (graph structure) between samples by aligning the labeled and unlabeled samples in the estimated subspace.
• We deeply integrate the adaptive graph learning with the proposed multitask regression framework in an iterative manner, making it possible for pseudo-labels to be gradually updated using the learned graph in each outer iteration.
• We also design a general solver that originates from the alternating direction method of multipliers (ADMM) optimizer for the solution of our proposed IMR method.

## 2 The proposed methodology

**Fig. 1:**
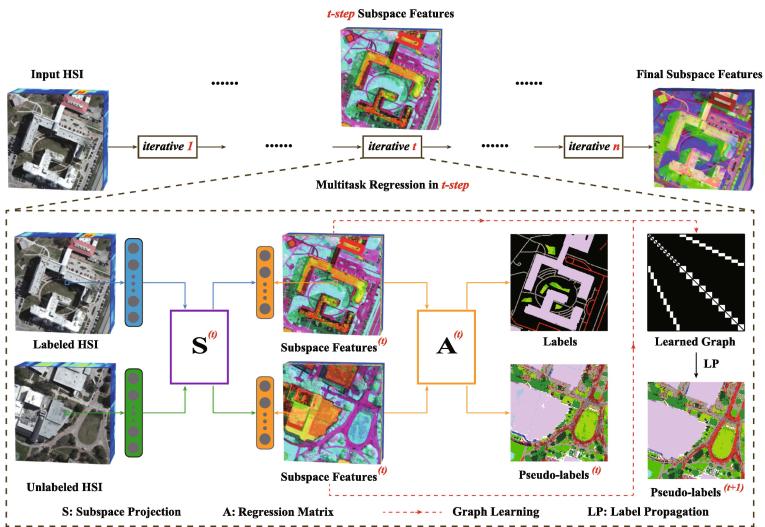
An overview of the proposed IMR framework. In fact, each iterative (*t*-*step*) starts with the input of labeled and unlabeled data and ends up the output of the subspace projections ($S^{\left(t\right)}$), regression matrix ($A^{\left(t\right)}$), and learned graph ($W^{\left(t\right)}$) aligning the labeled with unlabeled samples. With the *t*-*step* learned graph, the pseudo-labels (t+1) can be refined.

In this section, we start with a brief review of our model’s cornerstone, the JL framework, and then extend it to a variant of multitask learning by synchronously regressing the labeled and unlabeled data. We will further introduce the proposed iterative multitask regression (IMR) model by integrating the JL framework with the advanced graph learning technique, which more effectively propagates labels. Finally, an ADMM-based optimizer is used for the IMR solution. Fig. 1 illustrates the workflow of the proposed IMR method.

### 2.1 Review of the JL model

Let $X_{l}\in R^{d\times N}$ be the unfolded hyperspectral data with *d* bands by *N* pixels (or samples), and $Y_{l}\in R^{l\times N}$ be the corresponding one-hot encoded label matrix with *l* classes by *N* pixels. We model the original JL problem (Ji and Ye, 2009) as follows.

(1) $${\text{min}}_{A,S}\;\frac{1}{2}\|Y_{l}-{\mathrm{ASX}}_{l}{\|}_{\text{F}}^{2}+\frac{\alpha }{2}\|A{\|}_{\text{F}}^{2}\;\;s.t.\;\;{\mathrm{SS}}^{\text{T}}=I,$$

where $S\in R^{d_{\mathrm{sub}}\times N}$ and $A\in R^{l\times d_{\mathrm{sub}}}$ denote the subspace projection and the regression matrix linking the estimated subspace with label information, respectively, and $d_{\mathrm{sub}}$ represents the subspace dimension. $||•|{|}_{\text{F}}$ denotes the Frobenius norm and α is the regularization parameter.

Slightly different from the original JL, an improved model with manifold (graph) regularization is formulated by optimizing the following objective function.

(2) $${\text{min}}_{A,S}\;\frac{1}{2}\|Y_{l}-{\mathrm{ASX}}_{l}{\|}_{\text{F}}^{2}+\frac{\alpha }{2}\|A{\|}_{\text{F}}^{2}+\frac{\beta }{2}\text{tr}({\mathrm{SX}}_{l}L_{l}X_{l}^{\text{T}}S^{\text{T}})\;\;s.t.\;\;{\mathrm{SS}}^{\text{T}}=I,$$

where $L_{l}\in R^{N\times N}=D_{l}-W_{l}$ is the Laplacian matrix, $W_{l}\in R^{N\times N}$ is an adjacency matrix (graph), and $D_{l(\mathrm{ii})}=\sum_{i\neq j}W_{L(i,j)}$ is the corresponding degree matrix. The term tr denotes the trace of matrix parameterized by β. The JL-based models in Eqs. (1), (2) have been proven to be effectively solved with the ADMM optimizer (Hong et al., 2019b). Once the projection matrix S is learned, the subspace features can be computed by SX.

### 2.2 Iterative Multitask Regression (IMR)

Labeling in *Earth Vision* is extremely costly and time-consuming, as the remote sensing images have a larger-scale and more complex visual field. This leads to a limited number of labeled samples, which further hinders improvement of the model’s learning and generalization capability. To this end, we effectively utilize the information of unlabeled samples that are largely available by making a regression between the unlabeled samples and pseudo-labels in the form of multitask learning.

#### 2.2.1 Multitask regression with graph learning

In the multitask framework, we propose a learning-based graph regularization instead of a fixed graph artificially constructed with the known kernels (e.g., using Gaussian kernel function), in order to depict the connectivity (or similarity) between samples. Accordingly, a multitask regression framework is proposed for semi-supervised HDR by optimizing the following objective function.

(3) $${\text{min}}_{A,S,L}\left\{\begin{array}{cc} & \frac{\gamma }{2}\|Y_{l}-{\mathrm{ASX}}_{l}{\|}_{\text{F}}^{2}+\frac{1-\gamma }{2}\|Y_{\mathrm{pl}}-{\mathrm{ASX}}_{\mathrm{pl}}{\|}_{\text{F}}^{2}+\frac{\alpha }{2}\|A{\|}_{\text{F}}^{2}\\ & +\frac{\beta }{2}\text{tr}({\mathrm{SXLX}}^{\text{T}}S^{\text{T}})\\ & s.t.\;\;{\mathrm{SS}}^{\text{T}}=I,\;\;L=L^{\text{T}},\;\;L_{i,j,i\neq j}⪯0,\;\;L_{i,j,i=j}⪰0,\;\;\text{tr}(L)=s \end{array}\right\},$$

where $X_{\mathrm{pl}}\in R^{d\times M}$ and $Y_{\mathrm{pl}}\in R^{l\times M}$ denote unlabeled hyperspectral data and a one-hot encoded pseudo-label matrix, respectively, while $X=[X_{l},\;X_{\mathrm{pl}}]\in R^{d\times (N+M)}$ and $L\in R^{\left(N+M)\times (N+M\right)}$ is a joint Laplacian matrix. The term s>0 is a constant to control the scale. Furthermore, the two fidelity terms in multitask learning are balanced by a penalty parameter γ.

**Fig. 2:**
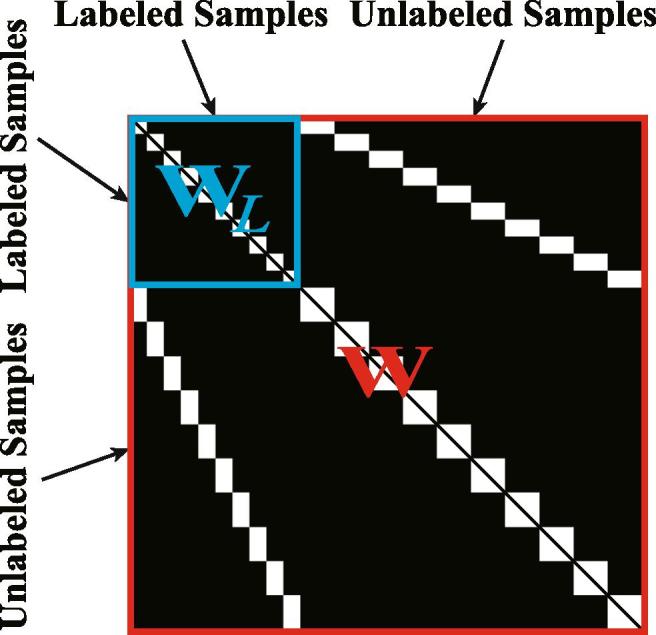
A showcase for joint adjacency matrix (W) (in ), where $W_{L}$ (in ) is a LDA-like graph constr.ucted by labels.

To solve (3) effectively, we rewrite the trace term as

(4) $$\text{tr}({\mathrm{SXLX}}^{\text{T}}S^{\text{T}})=\frac{1}{2}\text{tr}(\mathrm{WZ})=\frac{1}{2}\|W⊙Z{\|}_{1,1},$$

where $W\in R^{\left(N+M)\times (N+M\right)}$ is the to-be-learned joint adjacency matrix (see Fig. 2 in red). In W, the similarities between X can be measured by a *pair-wise distance matrix* ($Z\in R^{\left(2N+M)\times (2N+M\right)}$) on Euclidean space; this matrix can be computed by $Z_{i,j}=\|{\left(\mathrm{SX}\right)}_{i}-{\left(\mathrm{SX}\right)}_{j}{\|}^{2}$. Moreover, the operator ⊙ is interpreted as a *term-wise Schur-Hadamard* product.

By means of Eq. (4), optimizing problem (3) on a smooth manifold can be equivalently converted on a sparse graph as follows.

(5) $${\text{min}}_{A,S,W}\left\{\begin{array}{cc} & \frac{\gamma }{2}\|Y_{l}-{\mathrm{ASX}}_{l}{\|}_{\text{F}}^{2}+\frac{1-\gamma }{2}\|Y_{\mathrm{pl}}-{\mathrm{ASX}}_{\mathrm{pl}}{\|}_{\text{F}}^{2}+\frac{\alpha }{2}\|A{\|}_{\text{F}}^{2}+\frac{\beta }{4}\|W⊙Z{\|}_{1,1}\\ & \;s.t.\;\;{\mathrm{SS}}^{\text{T}}=I,\;\;W=W^{\text{T}},\;\;W_{i,j}⪰0,\;\;\|W{\|}_{1,1}=s \end{array}\right\}\text{.}$$

In Eq. (5), the $\|W⊙Z{\|}_{1,1}$ is specified as a *point-wise weighted* $\ell_{1}$*-norm* with respect to the variable of W, yielding a weighted sparsity.

Algorithm 1 Iterative Multitask Regression (IMR)

**Table:**

#### 2.2.2 Optimizing pseudo-labels with graph-based label propagation

In Eq. (3), the pseudo-labels are predicted by using a trained classifier, e.g., SVM or random forest. Although the model’s performance can be moderately improved through the use of unlabeled samples and pseudo-labels, yet the discrimination of the dimension-reduced HSI still remains limited by only regressing the static pseudo-labels. For this reason, the labels are dynamically propagated on the learned graph using GLP, when the model converges in each step^1^, aiming at iteratively refining or optimizing pseudo-labels, as illustrated in Fig. 1. The updated pseudo-labels together with the other inputs of $X_{l},X_{\mathrm{pl}}$, and $Y_{l}$ can be re-fed into the next round of model training, thus progressively improving the learning and generalization ability of the proposed multitask model.

### 2.3 Modal learning

Unlike the previous HDR methods following the graph embedding framework (Ma et al., 2010, Sun et al., 2014, Hong et al., 2017, Huang et al., 2019, Huang et al., 2019) that solve low-dimensional embedding as a problem of generalized eigenvalues decomposition (GED) (Yan et al., 2007), our model learning process is to iteratively and alternately optimize several convex subproblems with respect to the variables A,S, and W as well as to-be-updated $Y_{\mathrm{pl}}$ instead of directly solving the non-convex problem (5) by the separable strategy of the variables. An implementation of the proposed IMR is summarized in Algorithm 1. Such optimization strategy has been proven to be effective for solving the aforementioned issue (Bertsekas, 1997, Boyd et al., 2011) and successfully applied in many real cases (Ji and Ye, 2009, Hong et al., 2018, Hong et al., 2019b, Hong et al., 2019c).

#### 2.3.1 Learning regression matrix (A)

Intuitively, the optimization problem for solving the variable A is a Tikhonov-regularized least square regression, which is formulated as follows.

(6) $${\text{min}}_{A}\frac{\gamma }{2}\|Y_{l}-{\mathrm{ASX}}_{l}{\|}_{\text{F}}^{2}+\frac{1-\gamma }{2}\|Y_{\mathrm{pl}}-{\mathrm{ASX}}_{\mathrm{pl}}{\|}_{\text{F}}^{2}+\frac{\alpha }{2}\|A{\|}_{\text{F}}^{2}\text{.}$$

A closed-form solution of Eq. (6) is given by

(7) $$A=(\gamma Y_{l}H_{l}+(1-\gamma )Y_{\mathrm{pl}}H_{\mathrm{pl}})\times {\left(\gamma H_{l}H_{l}^{\text{T}}+(1-\gamma )H_{\mathrm{pl}}H_{\mathrm{pl}}^{\text{T}}+\alpha I\right)}^{-1},$$

where $H_{l}={\mathrm{SX}}_{l}$ and $H_{\mathrm{pl}}={\mathrm{SX}}_{\mathrm{pl}}$.

#### 2.3.2 Learning subspace projections (S)

The variable S can be estimated by solving the following optimization problem.

(8) $$\begin{array}{cc} & {\text{min}}_{S}\;\frac{\gamma }{2}\|Y_{l}-{\mathrm{ASX}}_{l}{\|}_{\text{F}}^{2}+\frac{1-\gamma }{2}\|Y_{\mathrm{pl}}-{\mathrm{ASX}}_{\mathrm{pl}}{\|}_{\text{F}}^{2}+\frac{\beta }{2}\text{tr}({\mathrm{SX}}_{l}L_{l}X_{l}^{\text{T}}S^{\text{T}})\\ & \;s.t.\;\;{\mathrm{SS}}^{\text{T}}=I\text{.} \end{array}$$

The orthogonality-constrained regression problem in Eq. (8) has been effectively solved by using an ADMM-based optimization algorithm (Hong et al., 2019b).

#### 2.3.3 Learning graph structure (W)

In the sub-problem, we learn the connectivity (or similarity) between samples from the data rather than using certain existing distance measurements. Therefore, the resulting optimization problem can be formulated as

(9) $${\text{min}}_{W}\;\frac{\beta }{4}\|W⊙Z{\|}_{1,1}\;\;s.t.\;W=W^{\text{T}},\;1/N_{k}⪰W_{i,j}⪰0,\;\|W{\|}_{1,1}=s,$$

whose solution has been obtained with an effective ADMM as well, as presented in Hong et al. (2019c). Please note that for those samples with labels, we construct a graph-based local discriminant analysis (LDA) (Belkin and Niyogi, 2003) in the place of the corresponding part in the learned graph W, as shown in Fig. 2. The LDA-like graph ($W_{L}$) can be expressed by

(10) $$W_{L(i,j)}=\left\{\begin{array}{cc} \frac{1}{N_{k}},\; & X_{i}\;\mathrm{and}\;X_{j}\;\mathrm{are}\;\mathrm{the}\;\mathrm{samples}\;\mathrm{belonging}\;\mathrm{to}\;\mathrm{the}\;k-\mathrm{th}\;\mathrm{class}\text{;}\\ 0\;,\; & \mathrm{otherwise}, \end{array}\right)$$

where $N_{k}$ denotes the number of samples belonging to *k*-th class.

#### 2.3.4 Updating pseudo-labels ($Y_{\mathrm{pl}}$)

**Fig. 3:**
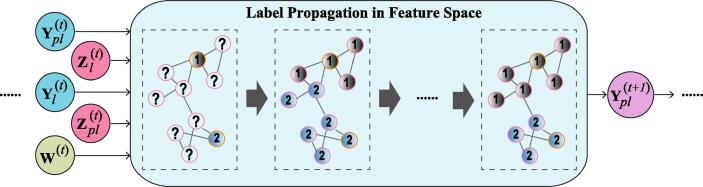
An illustration of label propagation used for updating the pseudo-labels, where $Z_{l}^{\left(t\right)}=S^{\left(t\right)}X_{l}$ and $Z_{\mathrm{pl}}^{\left(t\right)}=S^{\left(t\right)}X_{\mathrm{pl}}$ denote the low-dimensional feature representation for the labeled and unlabeled samples, respectively.

Given the labels ($Y_{l}$) and pseudo-labels ($Y_{\mathrm{pl}}^{\left(t\right)}$) of the t-*th* step, and the labeled ($X_{l}$) and unlabeled ($X_{\mathrm{pl}}$) samples, we can correspondingly learn the joint graph structure ($W^{\left(t\right)}$) in the t-*th* step from the t-*th* latent feature spaces ($Z^{\left(t\right)}$). The learned $W^{\left(t\right)}$ can then be further applied to infer the pseudo-labels of next step ($Y_{\mathrm{pl}}^{\left(t+1\right)}$) by LP, and then the updated pseudo-labels can be fed into a next-round model learning. This process is illustrated in Fig. 3. Please note that the model’s iteration will be suspended as long as the to-be-learned adjacency matrix W is not changed or the residual error (∊) between the current $W^{\left(t\right)}$ and the former step $W^{\left(t-1\right)}$ are close to zero (e.g., $10^{-6}$).

### 2.4 Convergence analysis and computational complexity

**Fig. 4:**
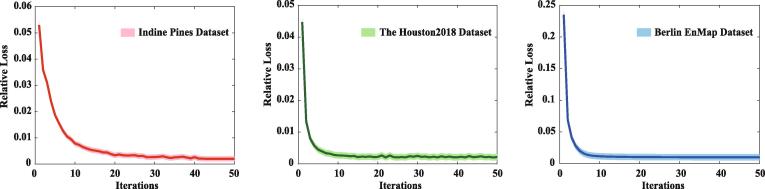
Convergence analysis of the proposed IMR method on three different datasets: Indine Pines, Houston2018, and Berlin EnMap. Note that the relative loss recorded in the convergence curve is obtained by averaging the loss values of multiple outer iterations in our proposed method.

Considering the non-convexity of Eq. (5) when all variables are considered simultaneously, a common and effective solution for the optimization problem is using a block coordinate descent (BCD) by alternatively optimizing each subproblem with respect to A,S, and W in an alternating strategy. The BCD algorithm has been guaranteed in theory to converge to a stationary point, if and only if each to-be-estimated variable in Eq. (5) can be exactly minimized (Bertsekas, 1997). Owing to the convexity in each independent task, a unique minimum can be ideally found in our case when the Lagrangian parameters used in ADMM are updated within finitely iterative steps (Boyd et al., 2011). The same or similar criterion has been successfully applied in various practical applications (Hong et al., 2017, Zhou et al., 2017, Xu et al., 2018b, Hong and Zhu, 2018). In addition, we also draw the convergence curves corresponding to the three used datasets, respectively, by recording the relative loss of objective function of Eq. (5) in each iteration, as shown in Fig. (4). One can be seen from the figure is that our model is able to fast reach the state of convergence with more or less 20 steps.

As observed in Section 2.3: **Model Learning**, the computational cost in our IMR model is mainly dominated by matrix products, where the most costly step lies in solving S, yielding an overall $O(d{\left(2N+M\right)}^{2}t)$ computational cost for Eq. (5).

## 3 Experiments

### 3.1 Data description

Three popular and promising HSI datasets – Indian Pines (Baumgardner et al., 2015), Houston2018 (Le Saux et al., 2018), and Berlin EnMap (Okujeni et al., 2016) – are used to assess the quantitative and qualitative performance of the IMR method, as briefly described below.

#### 3.1.1 Indian pines dataset

The hyperspectral scene located in the northwestern Indiana, USA, has been widely used in various HSI-related tasks, such as dimensionality reduction (Hong et al., 2016b, Hong et al., 2018) and classification (Dópido et al., 2012). It consists of 145×145 pixels with 220 spectral bands covering the wavelength from 400 nm to 2500 nm at intervals of 10 nm. There are 16 classes in the scene that are mostly vegetation, as detailed in Table 1 along with the number of training and test samples. Fig. 6 shows the false-color image of the studied scene as well as the distribution of training and test samples used in Ghamisi et al., 2014, Hong et al., 2018.

**Table 1:** Scene categories of the three HSI datasets used and the corresponding number of training and test samples for each class.

No.	IndianPine dataset			Houston2018 dataset			Berlin EnMap dataset		
	Class Name	TR	TE	Class Name	TR	TE	Class Name	TR	TE
1	CornNotill	50	1384	HealthyGrass	711	9088	Forest	656	11075
2	CornMintill	50	784	StressedGrass	3323	29179	Residential	825	56601
3	Corn	50	184	ArtificialTurf	171	513	Industrial	446	3735
4	GrassPasture	50	447	EvergreenTrees	954	12634	Low Plants	673	12006
5	GrassTrees	50	697	DeciduousTrees	350	4698	Soil	688	3040
6	HayWindrowed	50	439	BareEarth	664	3852	Allotment	415	2427
7	SoybeanNotill	50	918	Water	82	184	Commercial	367	4938
8	SoybeanMintill	50	2418	Residential	5375	34387	Water	184	1242
9	SoybeanClean	50	564	NonResidential	7794	215890	–	–	–
10	Wheat	50	162	Roads	3824	41986	–	–	–
11	Woods	50	1244	Sidewalks	1455	32547	–	–	–
12	BuildingsGrassTrees	50	330	Crosswalks	148	1368	–	–	–
13	StoneSteelTowers	50	45	Thoroughfares	4645	41713	–	–	–
14	Alfalfa	15	39	Highways	271	9578	–	–	–
15	GrassPastureMowed	15	11	Railways	391	6546	–	–	–
16	Oats	15	5	PavedParking	1271	10204	–	–	–
17	–	–	–	UnpavedParking	20	95	–	–	–
18	–	–	–	Cars	532	6046	–	–	–
19	–	–	–	Trains	154	5211	–	–	–
20	–	–	–	StadiumSeats	503	6321	–	–	–
									
	Total	695	9671	Total	9867	116123	Total	4254	95064

#### 3.1.2 Houston2018 dataset

This dataset is multi-modal data provided for the 2018 IEEE GRSS data fusion contest, where the HSI was acquired by an ITRES CASI 1500 sensor. The HSI, with dimensions of 601×2384×50, was collected from the wavelengths between 380 nm to 1050 nm at a ground sampling distance (GSD) of 1 m. This is a complex city scene with 20 challenging classes (see Fig. 7 and Table 1 for more details, including the specific training and test information). Note that we downsampled the ground truth map to the same GSD with the HSI by the nearest-neighbor-interpolation.

#### 3.1.3 Berlin EnMap dataset

The EnMap HSI with a GSD of 30 m was simulated by the corresponding HyMap data (Mueller et al., 2002) over a hybrid area that includes urban, rural, and vegetation in Berlin, Germany, this data is openly and freely available from the website^2^. This image consists of 797×220 pixels and 244 spectral bands in the wavelength ranging from 400 nm to 2500 nm. The ground truth in the scene is generated by the Haklay and Weber (2008) in the form of land cover and land use, and further refined and corrected by means of Google Earth. Table 1 lists the scene categories and the number of training and test samples, while the false-color image and corresponding distribution of training and test samples are given in Fig. 8.

### 3.2 Experimental configuration

#### 3.2.1 Evaluation metrics

With the input of different dimension-reduced features, we adopt the pixel-wise classification as a potential application for quantitative evaluation in terms of classification or recognition accuracy. More specifically, three commonly-used indices, *Overall Accuracy (OA)*, *Average Accuracy (AA)*, and *Kappa Coefficient (*κ*)*, are computed to quantify the experimental results using two simple but effective classifiers: nearest neighbor (NN) and linear SVM (LSVM). In our case, the two classifiers were selected because those more powerful classifiers (e.g., kernel SVM, random forest, deep neural network) tend to result in confusing evaluation, as it is unknown whether the performance improvement originates from either these advanced classifiers or the features itself.

#### 3.2.2 Comparison with state-of-the-art baselines

We evaluate the performance of the proposed IMR model visually and quantitatively in comparison with eight state-of-the-art baselines, including.

• **Non-HDR**: original spectral features (OSF);
• **Supervised HDR**: feature space discriminant analysis (FSDA) (Imani and Ghassemian, 2015), joint learning (JL) (Hong et al., 2019b);
• **Semi-supervised subspace learning for HDR**: semi-supervised local discriminant analysis (SELD) (Liao et al., 2013), collaborative discriminative manifold embedding (CDME) (Lv et al., 2017);
• **GLP-based semi-supervised HDR**: soft-label LDA (SL-LDA) (Zhao et al., 2014), semi-super- vised fisher local discriminant analysis (SSFLDA) (Wu and Prasad, 2018).

#### 3.2.3 Implementation preparation

The parameter settings for the algorithms play a key role in performance assessment. A common tactic for model selection is to run cross-validation on the training set. Following that, we conducted a 10-fold cross-validation to determine the optimal parameter combination for the different algorithms. In detail, there parameters that need to be tuned to maximize the classification performance on the training set were subspace dimension^3^ ($d_{\mathrm{sub}}$), selected from 5 to 50 at intervals of 5; the number of nearest neighbors (*k*); the standard deviation (σ) in SELD and SSLFDA, ranging from {10,20,…,50} and $\left\{10^{-2},10^{-1},10^{0},10^{1},10^{2}\right\}$, respectively; and the regularization parameters (e.g., α and β) in JL, CDME, and IMR in the range of $\left\{10^{-2},10^{-1},10^{0},10^{1},10^{2}\right\}$, while another regularization parameter γ in IMR can be selected from {0.1,0.2,…,0.9}. Moreover, initializing the adjacency matrix (W) and pseudo-labels ($Y_{\mathrm{pl}}$) in IMR is also an important factor in determining the model’s performance. We first predict the unlabeled samples using a pre-trained classifier on the training set; then the predicted results can be naturally input into the model as pseudo-labels. Likewise, the initialized W can be given by the labels and pseudo-labels. In addition, note that the clustering technique (e.g., K-means) is applied to handle the highly computational complexity caused by the large quantity of unlabeled samples during the process of model learning. As a trade-off, the number of cluster centers used in our case is approximately set to be the same as that of the training samples.

#### 3.2.4 The number of iterations in the proposed IMR

**Fig. 5:**
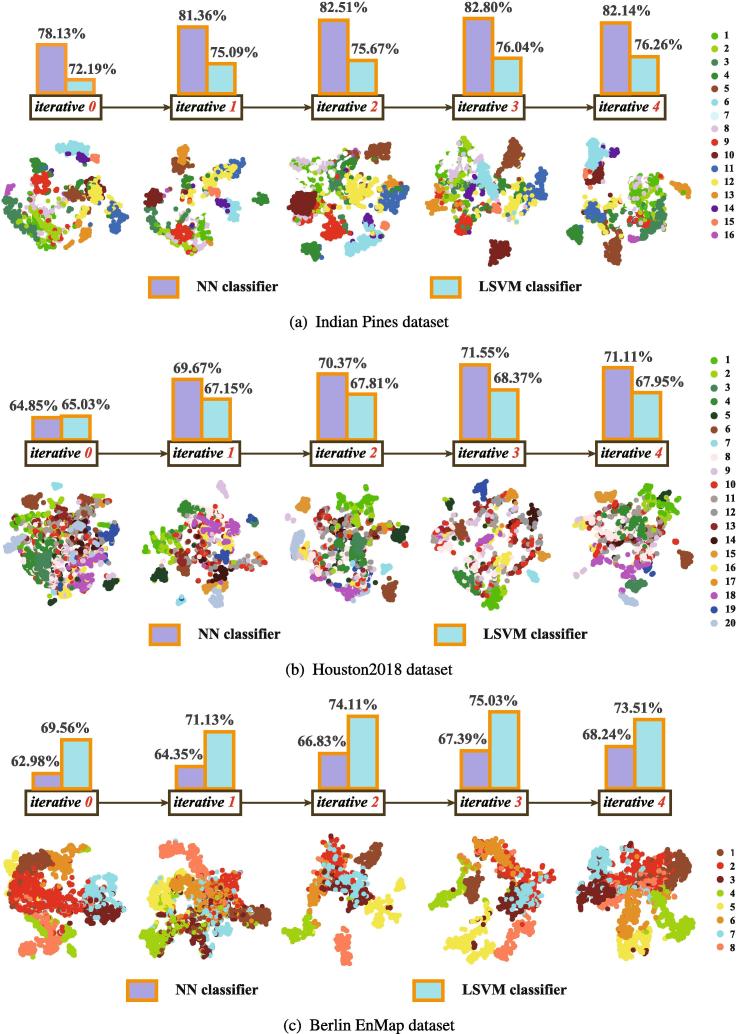
Visual and quantitative (OA) performance analysis with the different number of iterations in IMR on the three datasets.

According to the model’s stopping criteria in Algorithm 1, our IMR method generally converges to a desirable solution that corresponds to a well-learned adjacency matrix (W) out of three or four iterations. To support the results more effectively, we further investigate the effects of assigning a different number of iterations in IMR for the three datasets. Fig. 5 gives both visual and quantitative results with the increase of the IMR’s iterations^4^. Note that the IMR with *iterative 0* equivalently degrades to a version without label propagation. The OAs are clearly much lower without using an iterative strategy to update pseudo labels (*iterative 0*) than when using several iterations. Intuitively, this proves the superiority of the iterative strategy by gradually optimizing the pseudo-labels. It is worth noting, however, that the performance gain starts to slow down after two iterations and then remains essentially stable in the follow-up iterations, as the variable W is hardly changed any further. Similarly, for the different number of iterations, there is a consistent trend in the compactability of intra-class and the separability of inter-class. To summarize, we determine the number of iterations in the IMR to be 3 (IMR-3 for short); it will be used for comparison in the following experiments.

### 3.3 Results and analysis

#### 3.3.1 The Indian pines dataset

**Fig. 6:**
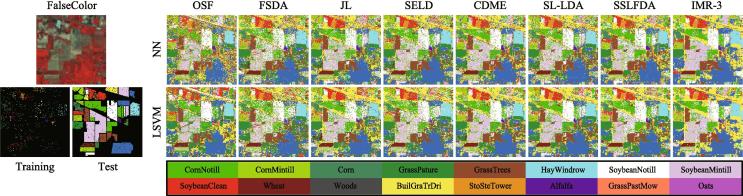
False-color image, the distribution of training and test samples as well as classification maps of the compared methods using two different classifiers on the Indian Pines dataset. (For interpretation of the references to colour in this figure legend, the reader is referred to the web version of this article.)

Fig. 6 presents the classification maps for different HDR compared methods using two classifiers on the Indian Pines dataset; Table 2 correspondingly lists the quantitative results obtained under the optimal parameter combination.

**Table 2:** Quantitative performance comparison among the different algorithms with the optimal parameters on the IndianPines dataset in terms of OA, AA, and κ as well as accuracy for each class. The best is shown in bold. Note that IMR-3 denotes the IMR with three iterations.

Methods	OSF (%)		FSDA (%)		JL (%)		SELD (%)		CDME (%)		SL-LDA (%)		SSLFDA (%)		IMR-3 (%)	
Parameter	*d*		*d*		(α,β,d)		(k,σ,d)		(α,β,d)		*d*		(k,σ,d)		(α,β,γ,d)	
	220		15		(0.01,0.01,20)		(10,0.1,15)		(0.01,0.01,20)		15		(5,0.1,15)		(0.01,0.1,0.8,20)	
Classifier	NN	LSVM	NN	LSVM	NN	LSVM	NN	LSVM	NN	LSVM	NN	LSVM	NN	LSVM	NN	LSVM
OA	65.89	64.12	64.14	63.67	76.89	71.51	72.09	69.52	74.63	71.41	70.93	73.20	75.26	72.67	**82.80**	76.04
AA	75.71	73.62	74.52	72.98	84.94	82.54	80.09	75.33	83.25	83.06	82.20	83.96	85.91	83.71	**86.27**	81.80
κ	0.6148	0.5974	0.5964	0.5912	0.7379	0.6785	0.6838	0.6543	0.7117	0.6773	0.6713	0.6980	0.7200	0.6915	**0.8033**	0.7266
																
Class1	51.66	57.15	51.45	49.86	66.47	64.60	63.80	58.02	59.47	56.79	57.73	64.09	70.23	65.46	**74.64**	**73.05**
**Class2**	**57.40**	**53.57**	**48.47**	**47.19**	**72.19**	**64.54**	**62.76**	**56.12**	**65.31**	**67.47**	**59.69**	**66.84**	**67.35**	**61.86**	**66.20**	**58.29**
**Class3**	**70.65**	**81.52**	**69.57**	**74.46**	**86.96**	**83.70**	**76.09**	**71.74**	**73.91**	**85.87**	**71.74**	**83.15**	**87.50**	**88.59**	**86.96**	**80.98**
**Class4**	**88.14**	**87.25**	**90.60**	**83.45**	**94.63**	**90.83**	**93.06**	**90.60**	**94.63**	**92.84**	**94.63**	**93.74**	**94.85**	**93.51**	**89.26**	**82.10**
**Class5**	**81.78**	**80.06**	**86.80**	**86.37**	**90.10**	**88.09**	**91.39**	**85.65**	**91.25**	**87.37**	**88.52**	**88.95**	**93.54**	**89.96**	**95.55**	**91.68**
**Class6**	**95.90**	**91.34**	**97.95**	**97.49**	**99.32**	**95.67**	**98.63**	**97.95**	**97.72**	**97.72**	**98.41**	**97.72**	**98.41**	**97.49**	**98.41**	**98.18**
**Class7**	**66.56**	**66.45**	**58.06**	**62.31**	**73.31**	**66.45**	**63.40**	**58.93**	**74.95**	**72.66**	**73.20**	**79.63**	**75.16**	**71.90**	**82.79**	**64.71**
**Class8**	**55.21**	**42.51**	**42.97**	**43.59**	**63.52**	**53.80**	**55.96**	**55.54**	**62.82**	**53.89**	**54.43**	**53.23**	**55.21**	**52.69**	**78.41**	**68.53**
**Class9**	**53.01**	**65.96**	**71.45**	**66.49**	**81.56**	**75.18**	**75.53**	**75.18**	**68.44**	**68.44**	**68.44**	**69.15**	**78.01**	**81.91**	**83.51**	**70.74**
**Class10**	**98.15**	**95.06**	**99.38**	**99.38**	**99.38**	**99.38**	**99.38**	**99.38**	**99.38**	**99.38**	**99.38**	**99.38**	**99.38**	**99.38**	**99.38**	**99.38**
**Class11**	**82.88**	**82.56**	**85.53**	**84.57**	**89.31**	**86.25**	**88.83**	**89.07**	**92.12**	**88.18**	**87.94**	**88.91**	**89.87**	**88.99**	**94.50**	**94.05**
**Class12**	**50.91**	**67.27**	**77.88**	**80.61**	**82.12**	**80.00**	**77.58**	**78.79**	**80.91**	**83.64**	**81.21**	**85.76**	**81.52**	**75.15**	**74.55**	**71.82**
**Class13**	**97.78**	**95.56**	**97.78**	**95.56**	**95.56**	**97.78**	**95.56**	**93.33**	**95.56**	**97.78**	**97.78**	**93.33**	**97.78**	**95.56**	**88.89**	**91.11**
**Class14**	**79.49**	**58.97**	**74.36**	**56.41**	**84.62**	**74.36**	**79.49**	**64.10**	**84.62**	**76.92**	**82.05**	**79.49**	**94.87**	**76.92**	**87.18**	**64.10**
**Class15**	**81.82**	**72.73**	**100.00**	**100.00**	**100.00**	**100.00**	**100.00**	**90.91**	**90.91**	**100.00**	**100.00**	**100.00**	**90.91**	**100.00**	**100.00**	**100.00**
**Class16**	**100.00**	**80.00**	**40.00**	**40.00**	**80.00**	**100.00**	**60.00**	**40.00**	**100.00**	**100.00**	**100.00**	**100.00**	**100.00**	**100.00**	**80.00**	**100.00**

Using the NN classifier, there is basically the same classification performance in OSF and FSDA. Despite an improved supervised criteria, FSDA still yields poor classification accuracy, since directly projecting the original data into a discriminative subspace with the limited amount of labeled samples is very challenging, especially when dealing with noisy data (e.g., HSI) with various spectral variabilities. Overall, the classification performance by considering the unlabeled samples is better than that without considering them. It should be noted, however, that inspired by latent subspace learning, the JL model dramatically outperforms FSDA (more than 10% improvement), but also improves the OAs of around 4%, 6%, 2%, and 1%, respectively, compared to those semi-supervised HDR approaches (SELD, CDME, SL-LDA, and SSLFDA). This intuitively indicates the superiority of the regression-based JL model for feature learning. Following the JL-like model, the proposed IMR framework achieves the best performance owing to the multitask learning framework, where the labeled and unlabeled samples can be jointly regressed, and to the iterative updating strategy of pseudo-labels. There is a similar trend in classification performance using the LSVM classifier, yet its performance is relatively weaker than those with the NN classifier. The possible reason for that is the few training samples available, further leading to the poor estimation of decision boundary for the SVM-like classifier learning.

Furthermore, we can observe from Table 2 that our IMR not only outperforms other HDR methods in terms of *OA*, *AA*, and κ, but it also obtains highly competitive results for each class, particularly for those classes with a relatively limited number of training samples in comparison with the number of test samples, such as *Corn-Notill*, *Grass-Trees*, *Soybean-Notill*, *Soybean-Mintill*, *Soybean-Clean*, and *Wheat*. This provides powerful evidence of the effectiveness of transferring the unlabeled samples to the learned subspace and the superiority of iteratively optimizing pseudo-labels.

#### 3.3.2 The Houston2018 dataset

**Fig. 7:**
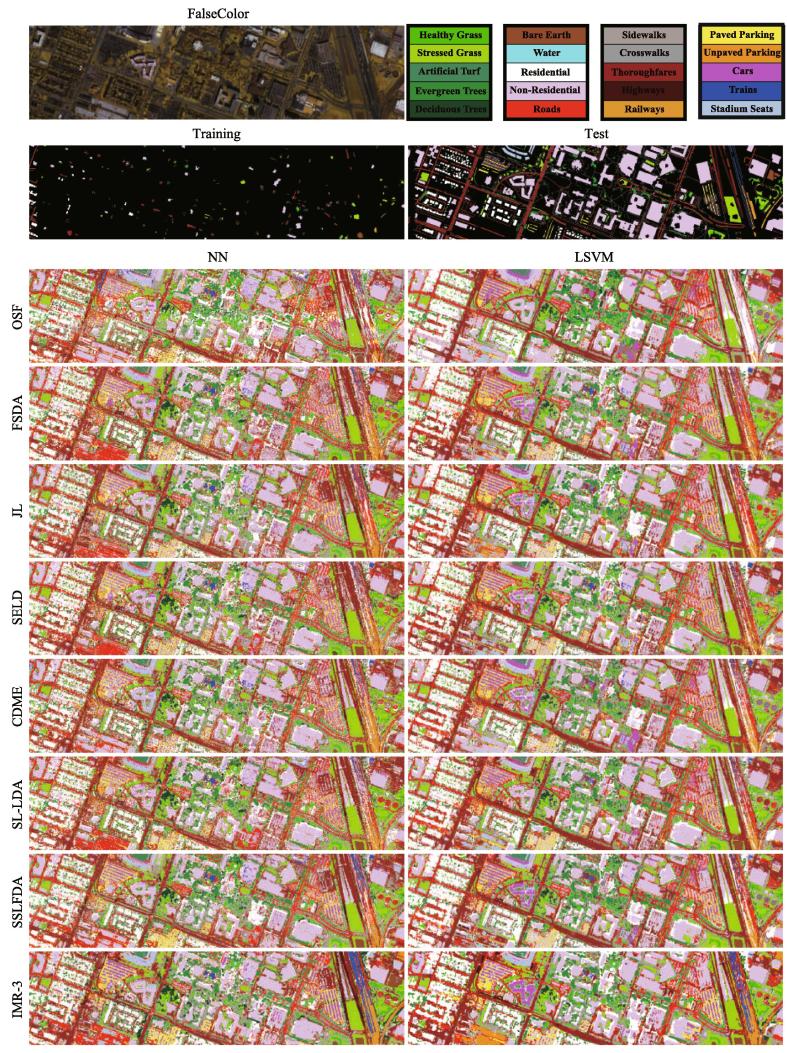
False-color image, the distribution of training and test samples as well as classification maps of compared methods using two different classifiers on the Houston2018 dataset. (For interpretation of the references to colour in this figure legend, the reader is referred to the web version of this article.)

Classification performance using the different low-dimensional feature representations is evaluated on the Houston2018 dataset both visually and quantitatively, as shown in Fig. 7 and listed in Table 3, respectively. The optimal parameters used for different compared methods are given in Table 3 as well. Likewise, due to more challenging categories in this scene and small-scale training set, the ability to classify the materials for the LSVM is limited. This might explain a phenomena in Table 3, that is, why the NN-based classifier, to some extent, performs better than the SVM-based one for many compared methods.

**Table 3:** Quantitative performance comparison among the different algorithms with the optimal parameters on the Houston2018 dataset in terms of OA, AA, and κ as well as accuracy for each class. The best is shown in bold. Note that IMR-3 denotes the IMR with three iterations.

Methods	OSF (%)		FSDA (%)		JL (%)		SELD (%)		CDME (%)		SL-LDA (%)		SSLFDA (%)		IMR-3 (%)	
Parameter	*d*		*d*		(α,β,d)		(k,σ,d)		(α,β,d)		*d*		(k,σ,d)		(α,β,γ,d)	
	50		19		(0.01,0.01,25)		(10,0.1,19)		(0.01,0.01,20)		19		(10,0.1,19)		(0.01,0.01,0.9,30)	
Classifier	NN	LSVM	NN	LSVM	NN	LSVM	NN	LSVM	NN	LSVM	NN	LSVM	NN	LSVM	NN	LSVM
OA	52.75	59.14	60.92	63.12	62.93	63.50	61.10	62.72	62.02	63.62	58.78	64.62	63.59	63.70	**71.55**	68.37
AA	46.77	42.97	55.15	50.85	56.72	50.87	55.21	50.71	54.81	51.07	53.26	52.65	58.51	52.94	**81.41**	67.07
κ	0.4232	0.4883	0.5161	0.5397	0.5390	0.5450	0.5187	0.5352	0.5261	0.5462	0.4921	0.5534	0.5506	0.5501	**0.6468**	0.6065
																
Class1	78.43	**89.50**	59.65	71.67	72.42	83.11	59.56	71.24	65.14	69.06	58.23	69.91	72.56	82.59	80.46	80.75
Class2	81.91	89.35	82.86	89.19	83.52	88.92	84.58	89.11	83.12	88.91	83.96	89.08	89.05	**91.46**	86.38	89.25
Class3	**100.00**	**100.00**	**100.00**	**100.00**	**100.00**	99.21	**100.00**	**100.00**	**100.00**	97.62	**100.00**	**100.00**	**100.00**	**100.00**	**100.00**	99.21
Class4	74.15	88.95	86.38	81.57	86.12	**91.12**	85.53	87.39	81.89	82.44	84.01	87.81	87.97	90.70	87.97	f90.90
Class5	14.94	9.68	30.05	15.03	27.33	12.14	28.86	15.79	27.84	14.60	27.25	19.78	28.10	16.64	**80.05**	30.05
Class6	11.32	12.00	13.45	12.00	19.28	17.26	15.25	12.00	20.18	15.70	12.89	12.00	12.00	12.00	**95.07**	31.17
Class7	60.00	31.11	60.00	57.78	60.00	55.56	60.00	55.56	60.00	55.56	84.44	60.00	60.00	51.11	**100.00**	95.56
Class8	77.97	85.46	85.46	87.89	84.63	86.37	85.54	86.92	81.33	88.84	85.90	85.70	87.29	**89.95**	86.67	89.37
Class9	56.49	63.84	65.25	68.01	67.23	67.52	64.88	67.45	68.41	68.27	62.54	71.53	65.07	65.58	**71.81**	68.84
Class10	37.17	39.19	39.79	46.20	43.24	49.03	40.53	45.15	39.07	**50.21**	38.07	46.77	48.92	47.65	45.00	49.92
Class11	31.97	34.29	34.42	40.81	38.91	39.14	35.94	37.45	35.72	39.67	31.33	36.21	43.78	41.38	43.17	**45.00**
Class12	5.95	0.00	6.25	0.00	10.12	0.30	5.65	0.00	6.55	0.30	5.65	0.00	17.86	0.00	**37.20**	1.79
Class13	48.04	65.54	57.83	59.12	63.10	63.52	60.34	62.03	59.57	62.04	58.51	64.73	65.54	69.59	67.30	**73.69**
Class14	10.89	0.00	18.48	9.43	20.98	4.01	15.52	7.76	16.40	8.18	18.56	4.80	16.52	8.09	**86.02**	29.24
Class15	8.10	1.35	62.92	34.50	37.75	18.85	54.51	29.65	67.77	32.17	40.64	34.19	31.00	24.80	**99.63**	81.09
Class16	52.11	42.82	70.81	73.87	76.58	73.17	74.02	70.96	62.02	66.74	64.73	58.19	85.17	73.75	**91.13**	85.13
Class17	88.89	0.00	72.22	22.22	88.89	16.67	77.78	27.78	72.22	33.33	61.11	61.11	**100.00**	44.44	**100.00**	88.89
Class18	48.59	72.46	63.98	73.15	67.98	76.54	59.01	76.26	56.38	77.50	59.49	62.66	72.81	73.43	**87.85**	70.95
Class19	23.55	0.93	35.60	29.03	35.44	19.61	34.21	25.71	34.05	25.41	30.35	30.89	43.78	29.27	**90.73**	69.88
Class20	24.98	32.89	57.69	45.57	50.85	55.43	62.46	46.08	58.51	44.95	57.63	57.69	42.69	46.26	**91.71**	70.56

More specifically, OSF yields a poor classification performance, due to the highly redundant spectral information and the sensitivity to noise. Unlike OSF that directly uses the original spectral features as the input features, FSDA and JL are apt to discriminate the materials due to the utilization of the label information. Further, taking the unlabeled samples into account is of great benefit in finding a better decision boundary, yielding a possible performance improvement, as shown in those subspace-based learning semi-supervised HDR methods (e.g., SELD, CDME). It is worth noting that the regression-based JL model is provided with nearly identical performance to those semi-supervised HDR approaches using both NN and LSVM classifiers, even though the powerful GLP is utilized (e.g., SL-LDA, SSLFDA). As expected, the performance of the IMR framework, which optimizes the pseudo-labels in an iterative fashion, is dramatically superior to that of others with the OA’s increase of approximately 8% (NN) and 5% (LSVM).

More intuitively, the proposed IMR performs better at identifying each material than other methods. In particular, when facing the extremely unbalanced sample distribution (see Table 1), our method gradually improves the quality of the pseudo-labels, thereby making the model develop a more powerful learning ability. Table 3 also reveals an interesting but unsurprising result: for those classes with a very limited number of training samples (e.g., *Deciduous Trees*, *Bare Earth*, *Water*, *Crosswalks*, *Highways*, *Unpaved Parking*, and *Stadium Seats*), the IMR makes a significant performance gain (an increase of at least 50% for these classes) with the aid of iterative pseudo-label learning.

#### 3.3.3 The Berlin EnMap dataset

**Fig. 8:**
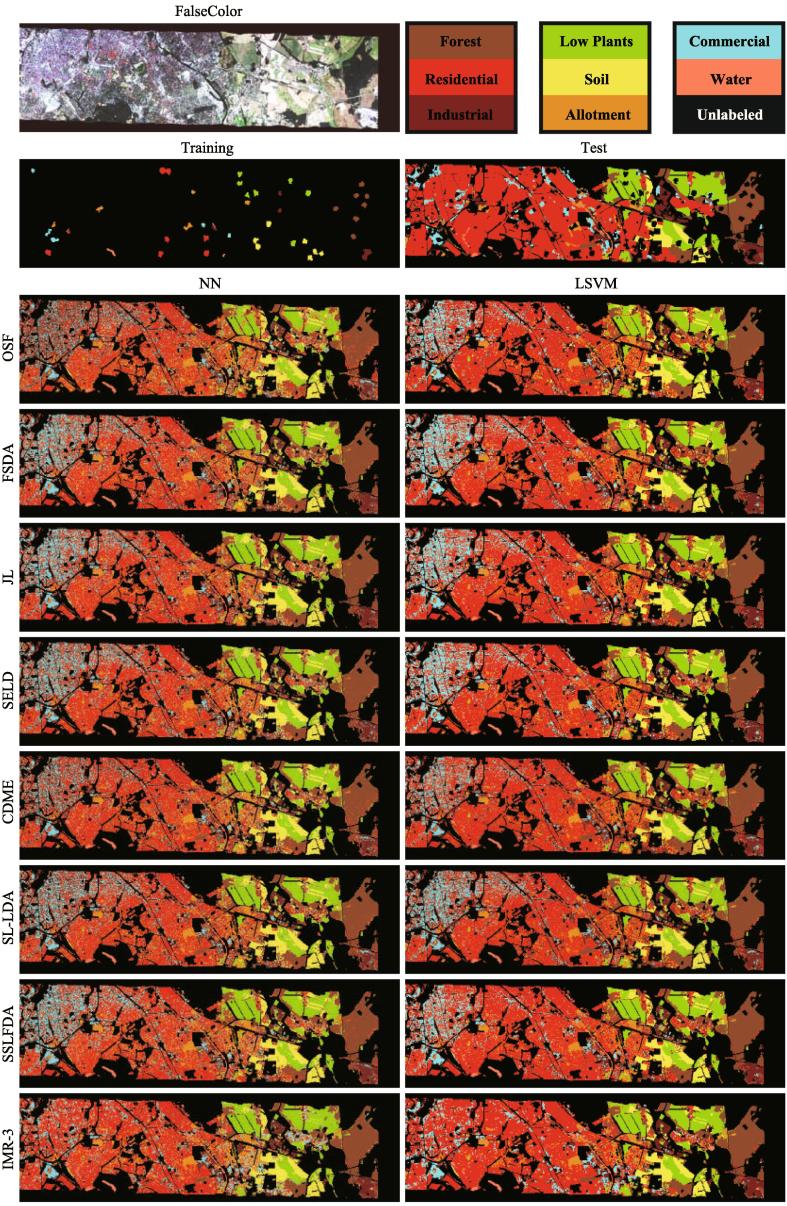
False-color image, the distribution of training and test samples as well as classification maps of compared methods using two different classifiers on the EnMap Berlin dataset. (For interpretation of the references to colour in this figure legend, the reader is referred to the web version of this article.)

For the Berlin EnMap dataset, the visual comparison of eight different algorithms in the form of classification maps is shown in Fig. 8. Table 4 details the comparison by means of three quantitative indices: *OA*, *AA*, and κ.

**Table 4:** Quantitative performance comparison among the different algorithms with the optimal parameters on the Berlin EnMap dataset in terms of OA, AA, and κ as well as accuracy for each class. The best is shown in bold. Note that IMR-3 denotes the IMR with three iterations.

Methods	OSF (%)		FSDA (%)		JL (%)		SELD (%)		CDME (%)		SL-LDA (%)		SSLFDA (%)		IMR-3 (%)	
Parameter	*d*		*d*		(α,β,d)		(k,σ,d)		(α,β,d)		*d*		(k,σ,d)		(α,β,γ,d)	
	244		7		(0.01,0.1,20)		(10,0.1,7)		(0.01,0.01,15)		7		(25,0.1,7)		(0.1,0.01,0.8,20)	
Classifier	NN	LSVM	NN	LSVM	NN	LSVM	NN	LSVM	NN	LSVM	NN	LSVM	NN	LSVM	NN	LSVM
OA	53.97	67.87	61.51	67.77	62.56	68.47	61.55	69.86	60.88	69.05	60.53	66.01	60.87	70.13	67.39	**75.03**
AA	57.47	66.04	64.61	65.98	64.71	65.90	63.79	65.76	62.88	65.13	63.87	65.34	65.96	67.36	69.05	**69.36**
κ	0.3781	0.5372	0.4711	0.5299	0.4821	0.5392	0.4702	0.5540	0.4621	0.5469	0.4619	0.5142	0.4668	0.5620	0.5411	**0.6222**
																
Class1	61.82	79.41	76.14	74.43	78.50	76.25	75.54	78.57	73.35	80.55	78.61	80.15	74.18	80.26	80.48	**81.91**
Class2	51.39	67.42	57.50	68.11	58.89	68.94	57.70	70.92	57.80	69.92	55.75	64.37	55.92	70.32	64.81	**77.61**
Class3	43.72	55.56	55.26	56.79	56.79	57.40	51.35	54.00	49.16	58.31	49.02	53.47	51.94	53.65	**61.95**	61.85
Class4	60.06	70.63	70.66	69.71	70.40	70.66	72.62	71.78	71.16	71.02	72.51	72.83	71.71	72.91	**74.76**	73.60
Class5	89.54	87.63	89.90	91.68	90.46	92.43	90.89	92.47	92.11	92.96	90.69	**93.36**	92.83	90.59	91.87	88.82
Class6	59.21	66.50	61.93	65.55	61.48	64.40	58.71	60.77	61.35	62.22	60.53	64.81	67.33	64.94	**68.44**	65.06
Class7	32.46	40.06	38.01	40.54	37.04	38.29	37.26	38.80	30.96	28.03	33.29	30.34	**42.89**	42.45	36.55	42.79
Class8	61.51	61.11	67.47	61.03	64.09	58.78	66.26	58.78	67.15	58.05	70.53	63.37	70.85	63.77	**73.51**	63.29

With a very high spectral dimension (244), OSF only holds a 53.97% accuracy when using the NN classifier. The performance of supervised HDR methods (SFDA and JL) is obviously superior to that of OSF, with an increase of at least 8% using the NN classifier. This reveals the importance of HDR in the follow-up hyperspectral data analysis. Furthermore, these methods exhibit balanced accuracies using the LSVM classifier, where JL shows a better classification performance owing to its well-designed architecture in the regression-based latent subspace learning. SELD learns the subspace projections by not only considering the label information but also computing the similarities between the unlabeled samples, yielding an effective semi-supervised low-dimensional embedding. However, the similarities between samples are usually measured by certain fixed functions, i.e., radial basis function (RBF), in the high-dimensional space, leading to poor robustness and ability to generalize. CDME implements an automatic similarity measurement by collaboratively representing the connectivity between the samples for the low-dimensional embedding. By the means of the soft (or pseudo) labels instead of using similarity measurement, SL-LDA and SSFLDA jointly use the labels and pseudo-labels to find a high discriminative subspace in a semi-supervised embedding approach.

Beyond the two subspace-based (SELD and CDME) and two GLP-based (SL-LDA and SSFLDA) semi-supervised strategies, we propose to iteratively optimize the pseudo-labels and feed them into a multitask regression framework in order to find a latent optimal subspace where the final decision boundary for different classes can be easily determined. On the other hand, our proposed IMR for each of the classes in the studied image exceeds the vast majority of compared methods except the material of *Commercial*, thereby further revealing the IMR’s advantages in low-dimensional representation learning.

### 3.4 Parameter sensitivity analysis

#### 3.4.1 On the regularization parameters

**Fig. 9:**
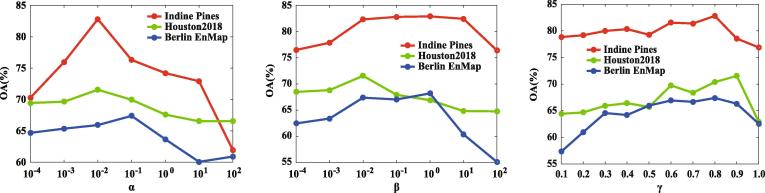
Sensitivity analysis on the regularization parameters (e.g., α,β, and γ) of the IMR in Eq. (5).

The quality of low-dimensional features extracted by the proposed IMR model is, to some extent, sensitive to the selection of three regularization parameters (α,β, and γ) as shown in Eq. (5). For this reason, we experimentally investigate the effects of different parameter setting in terms of OA via the NN classifier. The resulting analysis on the three datasets is quantified in Fig. 9, where the parameter combinations of (γ=0.8,α=0.01,β=0.1),(γ=0.9,α=0.01,β=0.01), and (γ=0.8,α=0.1,β=0.01) obtain the optimal classification performance on the test set for the Indine Pines dataset, Houston2018 dataset, and Berlin EnMap dataset, respectively. The results regrading the parameter setting are basically consistent with those obtained by cross-validation on the training set (see the Section 3.2.3: **Implementation Preparation**). Thus, the cross-validation strategy can be effectively used to determine the model’s parameters so that other researchers can produce the results for their tasks.

#### 3.4.2 On the subspace dimension

**Fig. 10:**
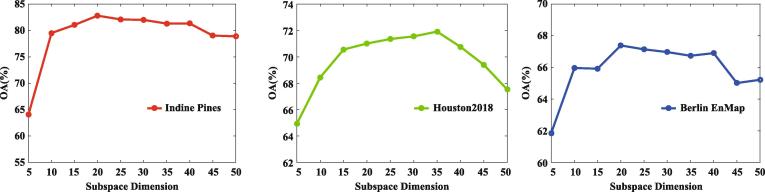
Sensitivity analysis on the subspace dimension in the proposed IMR method.

Apart from the regularization parameters, we analyze the performance gain in using the different subspace dimension of our IMR method, since a proper subspace dimension tends to reach a trade-off between discrimination and redundancy of the dimension-reduced product. For this purpose, the corresponding experiments are conducted by using the NN classifier to see the classification performance with the gradually-reducing dimension. As can be seen from Fig. 10, with the increase of subspace dimension, the IMR’s performance sharply increases to around 20 for first dataset, 30 for the second dataset, and 20 for the last dataset, respectively, then starts to reach a relatively stable state, and finally decreases with a slight perturbation when the subspace dimension is approaching to that of original spectral signature.

#### 3.4.3 On the training set size

**Fig. 11:**
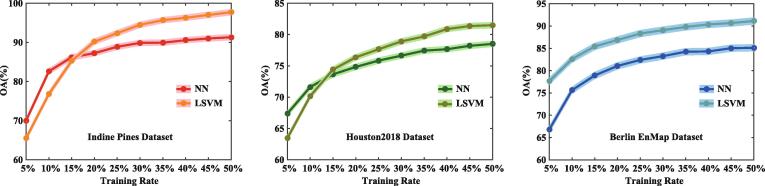
Sensitivity analysis to the size of training set using the NN and LVSM classifiers for the used three datasets.

Although the IMR adopts the semi-supervised learning strategy by jointly accounting for the labeled and unlabeled samples, yet the HDR’s performance is determined by the number of training samples to a great extent. This is, therefore, indispensable to investigate the sensitivity with an increasing size of training set. To highlight and emphasize the effectiveness and superiority of our proposed method in the HDR issue, we arrange the classification task by resetting the training set randomly selected from all labeled samples out of 10 run with the different proportions in the range of 5% to 50% at a 5% interval and the rest as the test set, and the average classification accuracies are reported by integrating the ten outputs in the end. Fig. 11 shows a similar trend in OAs with two classifiers (NN and LSVM) on the three different datasets, that is, the classification performance improves with the size of training set, faster in the early, and later basically stabilized. This also indicates that our semi-supervised method is not heavily dependent on a large-scale training set, which can hold a desirable and competitive performance in HDR, even when only small-scale labeled samples are used for training. On the other hand, we can observe an interesting conclusion on the first two datasets from the Fig. 11 that the NN classifier outperforms the LSVM one when the training samples are insufficient, e.g., less than around 15% of total samples. This could be well explained by the fact that LSVM is a learning-based classifier depending on the adequate samples for training an effective model, which is also supported by the experimental results yielding the higher OAs using the LSVM than those using the NN while using more training samples. Furthermore, with the increasing of training samples, the performance gain is prone to gradually become slow and meet the bottleneck, probably due to the lack of the spatial information modeling.

### 3.5 Computational cost in different methods

The experiments for HDR conducted by different methods are implemented for simulation on a laptop with the CPU i7-6700HQ (2.60 GHz) and a 32 GB random access memory (RAM). Herein, we assess the operational efficiency of the compared HDR approaches in terms of running time, as listed in Table 5.

**Table 5:** Time cost for the HDR of different methods on the three datasets.

Datasets	Time Cost (s)							
	OSF	FSDA	JL	SELD	CDME	SL-LDA	SSFLDA	IMR
Indine Pines	–	0.06	4.60	9.68	1.85	2.32	3.13	51.05
Houston2018	–	0.09	41.25	192.22	12.06	12.77	24.88	132.41
Berlin EnMap	–	0.22	48.81	57.81	10.82	11.48	25.20	75.72

In general, the running time of supervised HDR is much less than that of semi-supervised HDR, such as between supervised discriminant analysis (FSDA) and semi-supervised discriminant analysis (SELD, CDME, SL-LDA, and SSFLDA). The conclusion is just as much applicable to another group, that is, JL and our proposed IMR. Remarkably, although the newly-proposed IMR model seems to be operationally complex compared to other HDR methods, yet as it turns out, the IMR shows the computationally efficiency and the time cost is acceptable, mainly owing to the fast matrix-based computing power in regression-based techniques.

## 4 Conclusions

To facilitate the use of unlabeled samples effectively and efficiently, we propose a novel regression-based semi-supervised HDR model, called iterative multitask regression (IMR), which 1) simultaneously bridges the labeled and unlabeled samples with the labels and pseudo-labels in a multitask regression framework; and 2) progressively updates the pseudo-labels in an iterative fashion. This model provides us a new insight into the solutions of HDR-related problems. We conducted extensive experiments on three convincing and challenging HSI datasets, demonstrating that our method (IMR) is capable of extracting more discriminative features by allowing for the unlabeled samples and by optimizing the pseudo-labels.

It should be noted, however, that while there has been a desirable performance boost in IMR, it is still limited to working well only by linearly learning the low-dimensional feature representations for complex nonlinear cases. For this reason, our future work will address the HDR issue in a more complex scene and extend our framework to a nonlinear one with possible spatial information modeling.

## Footnotes

1 Given the inputs of $X_{l}$ and $X_{\mathrm{pl}}$ as well as $Y_{l}$ and $Y_{\mathrm{pl}}$, we estimate the variables of A,S, and L by solving problem (3). This process is defined as a “step” or in our case an “iteration”.

2 (http://doi.org/10.5880/enmap.2016.002).

3 For LDA-based approaches, e.g., FSDA, SELD, SL-LDA, and SSLFDA, the class number minus 1 is set to be $d_{\mathrm{sub}}$ (Martínez and Kak, 2001).

4 Here, we just showcase the results of four iterations, since in our case the model has usually converged around the number of iterations.
